# Intravenous thrombolysis for acute ischemic stroke associated with known left ventricular thrombus: safe or not?

**DOI:** 10.1186/s42466-022-00227-3

**Published:** 2022-12-22

**Authors:** Sofia Kitmeridou, Dimitrios Tsiptsios, Dimos Tsalkidis, Evlampia A. Psatha, Ioannis Iliopoulos, Nikolaos Aggelousis, Konstantinos Vadikolias

**Affiliations:** 1 Neurology Department, Democritus University of Thrace, Alexandroupolis, Greece; 2 Department of Physical Education and Sport Science, Democritus University of Thrace, Komotini, Greece

**Keywords:** Stroke, Intravenous thrombolysis, Left ventricular thrombus

## Abstract

Safety and efficacy data on intravenous thrombolysis (IVT) for acute ischemic stroke (AIS) in case of known left ventricular thrombus (LVT) are lacking. We present the case of a 35-year-old male with disabling AIS and known LVT that was treated successfully with intravenous alteplase. Apart from neurological improvement, post-procedural full thrombus lysis was also evident. Even though performing IVT in similar instances constitutes a difficult decision for physicians, it may be reasonable in the context of acute disabling stroke.

## Introduction

Up to today, straightforward guidelines on the proper management of acute ischemic stroke (AIS) associated with known left cardiac thrombus (LCT) are lacking. European Stroke Association (ESO) guidelines do not comment on whether intravenous thrombolysis (IVT) is contraindicated or not in such instances [1], whereas American Heart Association/American Stroke Association (AHA/ASA) guidelines propose that for patients with major AIS likely to produce severe disability and known LCT, treatment with intravenous (IV) alteplase may be reasonable, but for patients presenting with moderate AIS likely to produce mild disability and known LCT, treatment with IV alteplase is of uncertain net benefit [2].

## Case presentation

**Fig. 1:**
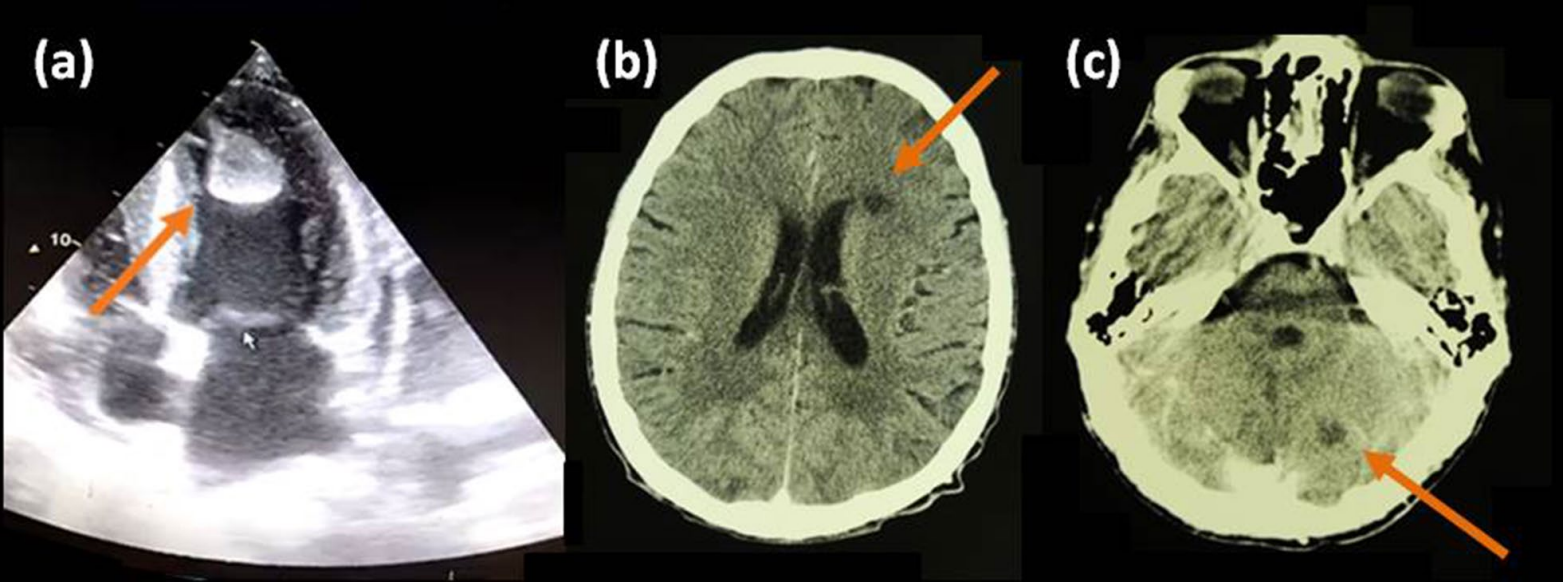
Pre-IVT TTE exhibiting LV thrombus (3.5 × 2.5 cm) with a narrow stalk (**a**) and CT scan demonstrating an ovoid hypodense lesion located in the vicinity of the left frontal horn (paraventricular white matter) (**b**) and a second similar hypodense lesion located in the left cerebellar hemisphere (**c**). Both CT lesions are imaging-wise compatible with lesions of a late subacute AIS-phase

A previously healthy 35-year-old Caucasian male was admitted to the coronary care unit of a nearby secondary care hospital due to acute dyspnea, bilateral lower limb oedema, night sweats, and elevated blood pressure (190/140 mmHg). Baseline electrocardiogram revealed sinus rhythm, whereas transthoracic echocardiogram (TTE) demonstrated: (a) left ventricular (LV) hypertrophy and dyskinesis, (b) 30% ejection fraction (EF), (c) a mobile LV thrombus (3.5 × 2.5 cm) with a narrow stalk (Fig. 1a) and (d) spontaneous echo contrast in the left cavities. Acute medication delivered included 40 mg furosemide IV twice daily, 5 mg nebivolol orally twice daily, and 4.500 IU tinzaparin subcutaneously once daily.

After 24 h of hospitalization, the patient developed acute consciousness disturbance, expressive aphasia, dysarthria, right central facial palsy and right upper extremity paresis (NIHSS: 6); thus, was transferred to the neurology department of our tertiary referral hospital.

**Fig. 2:**
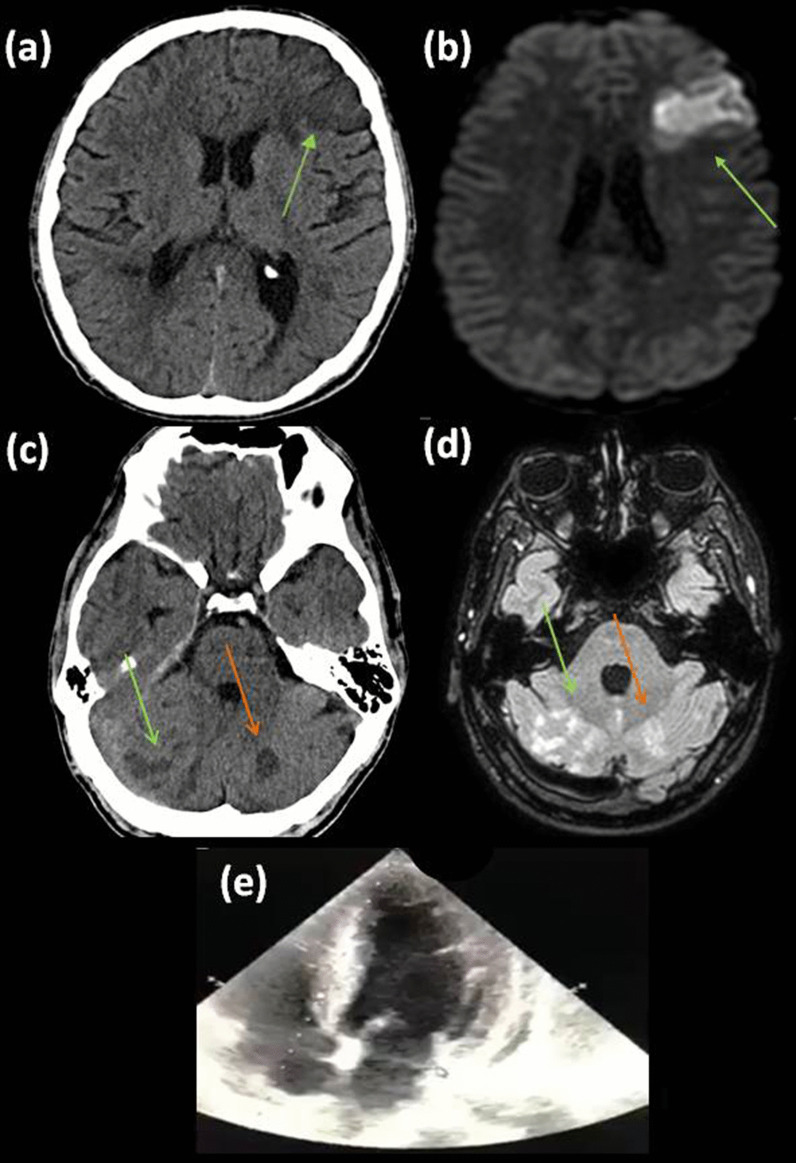
Post-IVT CT and DWI images demonstrating appearance of a new hypodense lesion (**a**) exhibiting true restriction (**b**) respectively, extending over an area irrigated by the superior trunk of the left MCA. Multiple new hypodense (CT)/high signal intensity lesions (FLAIR) within the right cerebellar hemisphere (PICA territory) and vermis (green arrows) and an older infarcted area on the left (orange arrows) (**c**, **d**). TTE exhibiting full LVT lysis (**e**)

Serum blood glucose and blood pressure levels at hospital admission were 103 mg/dL and 160/100 mmHg, respectively. Urgent brain CT revealed two preexisting infarcts, one near the left frontal horn and a second within the left cerebellar hemisphere, no evidence of new ischemic area (ASPECTS score: 10) (Fig. 1b, c). Four hours after symptom onset we proceeded with IVT with 70 mg alteplase (patient’s weight: 78 kg) that resulted in post-procedural NIHSS: 5. A follow-up CT scan performed 24 h afterwards revealed an acute left superior division middle cerebral artery stroke and punctate infarcts in both cerebellar hemispheres (Fig. 2a, c). Brain MRI recorded several acute and subacute infarcts in different arterial distributions (Fig. 2b, d).

Coronary angiogram performed four days afterwards was normal, whereas new TTE demonstrated EF of 45% and complete thrombus lysis (Fig. 2e). 1 week after symptom onset the patient was discharged home with NIHSS: 4 and mRS: 2 on a therapeutic dose of low molecular-weight heparin.

## Discussion

In our patient, urgent brain CT scan performed before IVT revealed two small infarcts of unknown time of occurrence. Due to the fact that he was neurologically asymptomatic, IVT was not contraindicated as according to ESO expert consensus statement IVT with alteplase is recommended in selected cases of recent infarcts, like those that are small and exhibit good clinical recovery [1].

The presence of those two infarcts in different vascular territories argued in favor of cardioembolic etiology and was consistent with the known large LVT. Proceeding with IVT in the presence of preexisting LVT is a difficult decision the physician must make due to the lack of data regarding the safety of the procedure [3]. Danger of complications from thrombus destabilization, breakup, or detachment, such as early recurrent ischemic stroke (ERIS) always exists. According to Georgiadis et al. in a series of 341 patients with AIS, 2 were diagnosed with ERIS and disintegration and subsequent scattering of cardiac or aortic thrombi was considered the underlying etiology [4]. Apart from ERIS, cases of myocardial, lower limb or systemic embolization complicating IVT are also mentioned [5, 6]. On the other hand, Derex et al. having studied 183 AIS patients, including 5 with known LCT, who were treated with IVT observed no early post-procedural systemic or cerebral embolism [7].

Thrombus characteristics that are associated with embolic events include protrusion into the LV cavity, mobility independent of myocardium, patient age > 68, thrombus area, length of the thrombi in the lumen, and LVT recurrence [8]. In our case, TTE revealed a mobile thrombus with stalk in the left ventricle with spontaneous echo contrast (“smoke”) indicating high risk of embolic events. However, according to AHA/ASA guidelines, IVT in the presence of LCT is not a contradiction in AIS likely to produce severe disability [9]. In our case, the possibility of residual severe post-stroke disability in a young patient due to expressive aphasia, dysarthria and loss of right hand dexterity led to the decision to proceed with IVT. Even though, small recurrent cerebral infarcts appeared, these were not clinically important. In contrast, significant post-IVT neurological improvement and functional independence were evident. Moreover, LVT resolved completely and EF improved.

Although our experience cannot be generalized and more studies are needed, our decision may be logical and in keeping with the comment of Tsivgoulis et al. that in potentially severe disability due to AIS, withholding IVT in case of known cardiac thrombus is not justified [10].

## Data Availability

Available upon reasonable request.

## Abbreviations

AIS: Acute ischemic stroke
AHA/ASA: American Heart Association/American Stroke Association
ASPECTS: Alberta Stroke Program Early CT Score
EF: Ejection fraction
ERIS: Early recurrent ischemic stroke
ESO: European Stroke Association
LV: Left ventricular
LCT: Left cardiac thrombus
LVT: Left ventricular thrombus
MCA: Middle cerebral artery
mg: Milligram
mRS: Modified Rankin Scale
NIHSS: National Institutes of Health Stroke Scale
IV: Intravenous
IVT: Intravenous thrombolysis
PICA: Posterior inferior cerebellar artery
TTE: Transthoracic echocardiogram
